# 4-(3-Carb­oxy-1-ethyl-6-fluoro-4-oxo-1,4-dihydroquinolin-7-yl)piperazin-1-ium 4-carb­oxy­benzoate–benzene-1,4-dicarb­oxy­lic acid (2/1)

**DOI:** 10.1107/S1600536811009524

**Published:** 2011-03-19

**Authors:** Shi-Wei Yan, Hai-Yan Chen, Guang-Ju Zhang, Qin Liao, Yan-Chen Liang

**Affiliations:** a College of Chemistry and Chemical Engineering, Southwest University, Chongqing 400715, People’s Republic of China

## Abstract

In the title compound, C_16_H_19_FN_3_O_3_^+^·C_8_H_5_O_4_^−^·0.5C_8_H_6_O_4_, the benzene-1,4-dicarb­oxy­lic acid mol­ecule is located on a centre of symmetry. In the crystal, the mol­ecules and ions are connected by inter­molecular C—H⋯O and O—H⋯O hydrogen bonds and π–π stacking inter­actions [with a centroid–centroid distance of 3.402 (2) Å], generating a three-dimensional supra­molecular structure.

## Related literature

For general background to the use of quinolones in the treatment of infections, see: Barbas *et al.* (2006 ▶); Basavoju *et al.*(2006 ▶); Xiao *et al.* (2005 ▶).
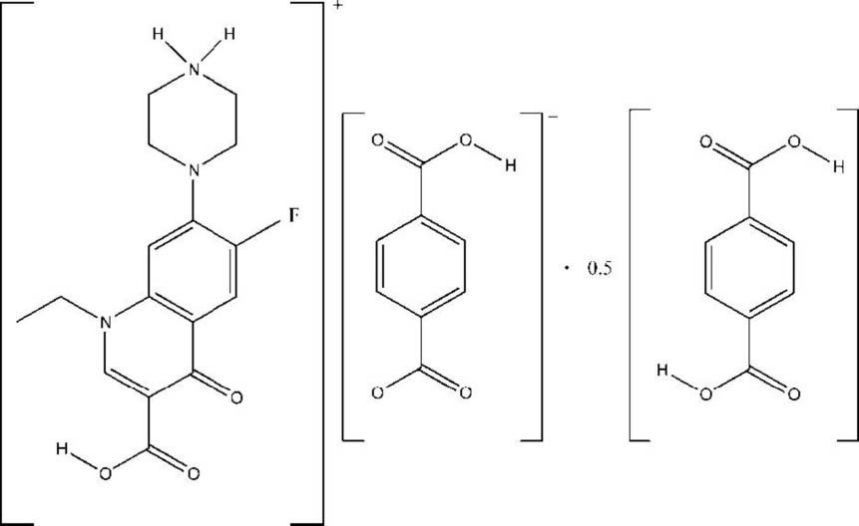


## Experimental

### 

#### Crystal data

C_16_H_19_FN_3_O_3_^+^·C_8_H_5_O_4_^−^·0.5C_8_H_6_O_4_
*M* *_r_* = 568.53
Triclinic, 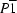

*a* = 9.8901 (15) Å
*b* = 10.2420 (16) Å
*c* = 13.665 (2) Å
α = 89.304 (2)°
β = 74.672 (2)°
γ = 71.677 (2)°
*V* = 1263.5 (3) Å^3^
*Z* = 2
Mo *K*α radiation
μ = 0.12 mm^−1^
*T* = 296 K
0.46 × 0.45 × 0.36 mm

#### Data collection

Bruker APEX CCD area-detector diffractometer
Absorption correction: multi-scan (*SADABS*; Sheldrick, 1996 ▶) *T*_min_ = 0.948, *T*_max_ = 0.959
10704 measured reflections
5143 independent reflections
4031 reflections with *I* > 2σ(*I*)
*R*_int_ = 0.024

#### Refinement

*R*[*F* ^2^ > 2σ(*F* ^2^)] = 0.042
*wR*(*F* ^2^) = 0.137
*S* = 1.00
5143 reflections
394 parameters
H atoms treated by a mixture of independent and constrained refinement
Δρ_max_ = 0.26 e Å^−3^
Δρ_min_ = −0.28 e Å^−3^

### 

Data collection: *SMART* (Bruker, 2001 ▶); cell refinement: *SAINT* (Bruker, 2001 ▶); data reduction: *SAINT*; program(s) used to solve structure: *SHELXS97* (Sheldrick, 2008 ▶); program(s) used to refine structure: *SHELXL97* (Sheldrick, 2008 ▶); molecular graphics: *SHELXTL-Plus* (Sheldrick, 2008 ▶); software used to prepare material for publication: *SHELXL97*.

## Supplementary Material

Crystal structure: contains datablocks I, global. DOI: 10.1107/S1600536811009524/ff2003sup1.cif

Structure factors: contains datablocks I. DOI: 10.1107/S1600536811009524/ff2003Isup2.hkl

Additional supplementary materials:  crystallographic information; 3D view; checkCIF report

## Figures and Tables

**Table 1:** Hydrogen-bond geometry (Å, °)

*D*—H⋯*A*	*D*—H	H⋯*A*	*D*⋯*A*	*D*—H⋯*A*
C15—H15*B*⋯O1^i^	0.97	2.38	3.274 (2)	153
O5—H5*B*⋯O7^ii^	0.97 (3)	1.70 (3)	2.6648 (15)	169 (2)

Symmetry codes: (i) 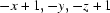; (ii) 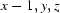.

**Fig. 1.**
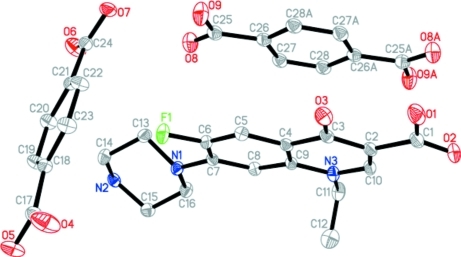
The structure of 1. Displacement ellipsoids are drawn at the 30% probability level. H atoms havebeen omitted for clarity [Symmetry code: -x+2, -y, -z+1].

## supplementary crystallographic information

### Comment

Norfloxacin [1–Ethyl–6–fluoro–1,4–dihydro–4–oxo–7–(piperazin–1–yl)quinoline–3–carbo–xylic acid] is member of a class of quinolones used to treat infections (Xiao *et al.*, 2005; Barbas *et al.*, 2006; Basavoju *et al.*, 2006). In this paper,the structure of the title compound, 1, is described (Fig. 1). In compound 1, benzene-1,4-dicarboxylic acid is located on the centre of symmetry. The molecules and the ions are linked by intermolecular C—H···O and O—H···O hydrogen-bonding interactions (C···O = 3.273 (2) Å, O···O = 2.6648 (18) Å) and π—π stacking between the benzene ring of [H_2_norf]^+^ and aromatic ring of 1,4-H_2_bdc with the centroid-centroid distance of 3.402 (2) Å, generating a three-dimensional supramolecular structure.

### Experimental

A mixture of Mn(CH_3_COO)_2_.4H_2_O (0.061 g, 0.25 mmol), Norfloxacin (0.080 g, 0.25 mmol) and distilled water (10 ml) was stirred for 20 min. in air. The mixture was then transferred to a 23 ml Teflon-lined hydrothermal bomb. The bomb was kept at 393 K for 96 h under autogenous pressure. Upon cooling, yellow block of 1 were obtained from the reaction mixture.

### Refinement

The H atoms bonded to C atoms were positioned geometrically and refined using a riding model approximation [aromatic C—H = 0.93 Å, aliphatic C—H = 0.97 Å], with *U*_iso_(H) = 1.2–1.5 *U*_eq_(C). The H on N atoms were located in a difference Fourier map, and refined with distances restraint of N—H = 0.90 Å–0.95 Å, and with *U*_iso_(H) = 1.5–1.7 *U*_eq_(N). The H atoms bonded to O atoms were located in a difference Fourier maps and with *U*_iso_(H) = 1.4, 1.9 and 2.1 *U*_eq_(O) for carboxyl groups of [H_2_norf]^+^, [1,4-Hbdc]^-^ and 1,4-H_2_bdc, respectively. The O—H bonds are 0.87 Å, 0.97 Å and 1.01 Å in carboxyl groups of [H_2_norf]^+^,[1,4–Hbdc]^-^ and 1,4–H_2_bdc.

### Crystal data

**Table:**

C_16_H_19_FN_3_O_3_^+^·C_8_H_5_O_4_^−^·0.5C_8_H_6_O_4_	*Z* = 2
*M**_r_* = 568.53	*F*(000) = 594
Triclinic, *P*1	*D*_x_ = 1.494 Mg m^−^^3^
Hall symbol: -P 1	Mo *K*α radiation, λ = 0.71073 Å
*a* = 9.8901 (15) Å	Cell parameters from 10704 reflections
*b* = 10.2420 (16) Å	θ = 2.5–26.4°
*c* = 13.665 (2) Å	µ = 0.12 mm^−^^1^
α = 89.304 (2)°	*T* = 296 K
β = 74.672 (2)°	Block, yellow
γ = 71.677 (2)°	0.46 × 0.45 × 0.36 mm
*V* = 1263.5 (3) Å^3^	

### Data collection

**Table:**

Bruker APEX CCD area-detector diffractometer	5143 independent reflections
Radiation source: fine-focus sealed tube	4031 reflections with *I* > 2σ(*I*)
graphite	*R*_int_ = 0.024
φ and ω scans	θ_max_ = 26.4°, θ_min_ = 2.5°
Absorption correction: multi-scan (*SADABS*; Sheldrick, 1996)	*h* = −12→12
*T*_min_ = 0.948, *T*_max_ = 0.959	*k* = −12→12
10704 measured reflections	*l* = −17→17

### Refinement

**Table:**

Refinement on *F*^2^	Primary atom site location: structure-invariant direct methods
Least-squares matrix: full	Secondary atom site location: difference Fourier map
*R*[*F*^2^ > 2σ(*F*^2^)] = 0.042	Hydrogen site location: inferred from neighbouring sites
*wR*(*F*^2^) = 0.137	H atoms treated by a mixture of independent and constrained refinement
*S* = 1.00	*w* = 1/[σ^2^(*F*_o_^2^) + (0.095*P*)^2^] where *P* = (*F*_o_^2^ + 2*F*_c_^2^)/3
5143 reflections	(Δ/σ)_max_ < 0.001
394 parameters	Δρ_max_ = 0.26 e Å^−^^3^
0 restraints	Δρ_min_ = −0.28 e Å^−^^3^

### Special details

**Table:**

Geometry. All e.s.d.'s (except the e.s.d. in the dihedral angle between two l.s. planes) are estimated using the full covariance matrix. The cell e.s.d.'s are taken into account individually in the estimation of e.s.d.'s in distances, angles and torsion angles; correlations between e.s.d.'s in cell parameters are only used when they are defined by crystal symmetry. An approximate (isotropic) treatment of cell e.s.d.'s is used for estimating e.s.d.'s involving l.s. planes.
Refinement. Refinement of *F*^2^ against ALL reflections. The weighted *R*-factor *wR* and goodness of fit *S* are based on *F*^2^, conventional *R*-factors *R* are based on *F*, with *F* set to zero for negative *F*^2^. The threshold expression of *F*^2^ > σ(*F*^2^) is used only for calculating *R*-factors(gt) *etc*. and is not relevant to the choice of reflections for refinement. *R*-factors based on *F*^2^ are statistically about twice as large as those based on *F*, and *R*- factors based on ALL data will be even larger.

### Fractional atomic coordinates and isotropic or equivalent isotropic displacement parameters (Å^2^)

**Table:**

	*x*	*y*	*z*	*U*_iso_*/*U*_eq_	
F1	0.63577 (11)	0.18180 (9)	0.27698 (7)	0.0431 (3)	
C1	0.63370 (16)	−0.02848 (19)	0.79008 (11)	0.0389 (4)	
C2	0.65495 (15)	−0.05532 (16)	0.68003 (10)	0.0307 (3)	
C3	0.63043 (15)	0.05539 (15)	0.61573 (11)	0.0290 (3)	
C4	0.66237 (14)	0.01485 (14)	0.50870 (10)	0.0258 (3)	
C5	0.64110 (15)	0.11621 (15)	0.43912 (10)	0.0275 (3)	
H5A	0.6076	0.2090	0.4619	0.033*	
C6	0.66906 (15)	0.07966 (14)	0.33949 (10)	0.0276 (3)	
C7	0.72669 (15)	−0.05884 (15)	0.29813 (10)	0.0264 (3)	
C8	0.75114 (15)	−0.15922 (15)	0.36672 (10)	0.0277 (3)	
H8A	0.7915	−0.2517	0.3427	0.033*	
C9	0.71625 (14)	−0.12410 (14)	0.47136 (10)	0.0258 (3)	
C10	0.70628 (16)	−0.18915 (16)	0.63933 (11)	0.0320 (3)	
H10A	0.7198	−0.2588	0.6836	0.038*	
C11	0.79437 (18)	−0.37552 (16)	0.50584 (12)	0.0397 (4)	
H11A	0.8377	−0.4277	0.5560	0.048*	
H11B	0.8717	−0.3910	0.4423	0.048*	
C12	0.6737 (2)	−0.42777 (18)	0.49078 (15)	0.0524 (5)	
H12A	0.7149	−0.5242	0.4686	0.079*	
H12B	0.6316	−0.3776	0.4403	0.079*	
H12C	0.5979	−0.4147	0.5539	0.079*	
C13	0.82815 (18)	−0.02111 (17)	0.11838 (11)	0.0367 (4)	
H13A	0.9339	−0.0663	0.1060	0.044*	
H13B	0.8050	0.0741	0.1427	0.044*	
C14	0.78474 (18)	−0.02599 (16)	0.02080 (11)	0.0377 (4)	
H14A	0.6795	0.0219	0.0328	0.045*	
H14B	0.8381	0.0201	−0.0302	0.045*	
C15	0.74848 (17)	−0.24840 (16)	0.06232 (11)	0.0353 (4)	
H15A	0.7813	−0.3450	0.0383	0.042*	
H15B	0.6419	−0.2125	0.0739	0.042*	
C16	0.78617 (19)	−0.23578 (15)	0.16120 (11)	0.0363 (4)	
H16A	0.7325	−0.2810	0.2127	0.044*	
H16B	0.8913	−0.2813	0.1517	0.044*	
C17	0.17400 (16)	0.50636 (16)	0.20944 (11)	0.0330 (3)	
C18	0.33786 (15)	0.48186 (14)	0.18335 (11)	0.0290 (3)	
C19	0.43591 (16)	0.39424 (16)	0.10056 (12)	0.0350 (3)	
H19A	0.4000	0.3534	0.0571	0.042*	
C20	0.58733 (16)	0.36724 (16)	0.08232 (11)	0.0344 (3)	
H20A	0.6523	0.3088	0.0264	0.041*	
C21	0.64275 (15)	0.42664 (14)	0.14690 (11)	0.0281 (3)	
C22	0.54455 (16)	0.51661 (16)	0.22799 (12)	0.0348 (3)	
H22A	0.5804	0.5588	0.2707	0.042*	
C23	0.39284 (16)	0.54453 (16)	0.24616 (12)	0.0356 (4)	
H23A	0.3278	0.6056	0.3007	0.043*	
C24	0.80895 (16)	0.38823 (15)	0.12883 (12)	0.0321 (3)	
C25	0.97781 (16)	0.15488 (18)	0.32394 (12)	0.0371 (4)	
C26	0.98985 (15)	0.07643 (17)	0.41574 (11)	0.0330 (3)	
C27	1.04199 (16)	−0.06690 (17)	0.40371 (12)	0.0354 (4)	
H27A	1.0697	−0.1117	0.3392	0.042*	
C28	1.05276 (16)	−0.14308 (18)	0.48721 (12)	0.0356 (4)	
H28	1.087 (2)	−0.246 (2)	0.4790 (14)	0.054 (5)*	
N1	0.74725 (14)	−0.09079 (12)	0.19528 (9)	0.0306 (3)	
N2	0.81977 (15)	−0.17166 (14)	−0.01670 (9)	0.0327 (3)	
H2A	0.923 (2)	−0.2219 (19)	−0.0393 (13)	0.049 (5)*	
H2B	0.785 (2)	−0.169 (2)	−0.0715 (15)	0.055 (5)*	
N3	0.73831 (13)	−0.22629 (12)	0.54007 (9)	0.0305 (3)	
O1	0.60295 (14)	0.10171 (15)	0.82150 (9)	0.0489 (3)	
H1A	0.595 (3)	0.154 (3)	0.760 (2)	0.104 (9)*	
O2	0.64619 (14)	−0.11994 (15)	0.84810 (9)	0.0522 (3)	
O3	0.58611 (12)	0.18033 (11)	0.64870 (8)	0.0391 (3)	
O4	0.08777 (13)	0.57308 (16)	0.28487 (10)	0.0622 (4)	
O5	0.13363 (12)	0.44329 (13)	0.14372 (9)	0.0466 (3)	
H5B	0.030 (3)	0.448 (2)	0.1690 (17)	0.087 (7)*	
O6	0.88873 (12)	0.30848 (14)	0.05353 (9)	0.0533 (3)	
O7	0.85565 (11)	0.43952 (11)	0.19277 (9)	0.0379 (3)	
O8	1.01763 (14)	0.10184 (13)	0.23849 (9)	0.0520 (3)	
O9	0.91792 (16)	0.28979 (13)	0.34610 (10)	0.0565 (4)	
H9A	0.903 (3)	0.334 (2)	0.2932 (18)	0.080 (8)*	

### Atomic displacement parameters (Å^2^)

**Table:**

	*U*^11^	*U*^22^	*U*^33^	*U*^12^	*U*^13^	*U*^23^
F1	0.0595 (6)	0.0329 (5)	0.0350 (5)	−0.0096 (4)	−0.0165 (4)	0.0123 (4)
C1	0.0302 (8)	0.0629 (11)	0.0270 (8)	−0.0185 (8)	−0.0093 (6)	0.0033 (8)
C2	0.0241 (7)	0.0454 (9)	0.0242 (7)	−0.0122 (6)	−0.0079 (6)	0.0031 (6)
C3	0.0215 (6)	0.0374 (8)	0.0271 (7)	−0.0080 (6)	−0.0069 (5)	−0.0025 (6)
C4	0.0208 (6)	0.0323 (8)	0.0245 (7)	−0.0080 (6)	−0.0069 (5)	0.0007 (6)
C5	0.0259 (7)	0.0254 (7)	0.0295 (7)	−0.0062 (6)	−0.0071 (6)	−0.0001 (6)
C6	0.0281 (7)	0.0284 (7)	0.0284 (7)	−0.0092 (6)	−0.0113 (6)	0.0079 (6)
C7	0.0265 (7)	0.0333 (8)	0.0221 (7)	−0.0127 (6)	−0.0075 (5)	0.0013 (6)
C8	0.0280 (7)	0.0262 (7)	0.0266 (7)	−0.0058 (6)	−0.0072 (6)	−0.0001 (5)
C9	0.0218 (6)	0.0307 (7)	0.0246 (7)	−0.0077 (6)	−0.0073 (5)	0.0043 (6)
C10	0.0291 (7)	0.0420 (9)	0.0265 (7)	−0.0106 (6)	−0.0114 (6)	0.0087 (6)
C11	0.0431 (9)	0.0297 (8)	0.0338 (8)	0.0027 (7)	−0.0076 (7)	0.0049 (6)
C12	0.0648 (12)	0.0311 (9)	0.0544 (11)	−0.0102 (8)	−0.0109 (9)	−0.0027 (8)
C13	0.0433 (9)	0.0441 (9)	0.0285 (8)	−0.0239 (7)	−0.0079 (6)	0.0049 (6)
C14	0.0460 (9)	0.0390 (9)	0.0270 (8)	−0.0145 (7)	−0.0073 (7)	0.0066 (6)
C15	0.0378 (8)	0.0392 (9)	0.0290 (8)	−0.0145 (7)	−0.0066 (6)	−0.0042 (6)
C16	0.0504 (9)	0.0330 (8)	0.0267 (8)	−0.0152 (7)	−0.0103 (7)	0.0006 (6)
C17	0.0268 (7)	0.0366 (8)	0.0350 (8)	−0.0092 (6)	−0.0087 (6)	0.0019 (6)
C18	0.0255 (7)	0.0310 (7)	0.0320 (8)	−0.0101 (6)	−0.0090 (6)	0.0048 (6)
C19	0.0310 (8)	0.0404 (9)	0.0365 (8)	−0.0125 (7)	−0.0126 (6)	−0.0041 (7)
C20	0.0291 (8)	0.0369 (8)	0.0339 (8)	−0.0078 (6)	−0.0066 (6)	−0.0035 (6)
C21	0.0251 (7)	0.0276 (7)	0.0341 (8)	−0.0099 (6)	−0.0106 (6)	0.0080 (6)
C22	0.0305 (8)	0.0378 (8)	0.0388 (8)	−0.0130 (6)	−0.0116 (6)	−0.0044 (7)
C23	0.0295 (8)	0.0366 (8)	0.0371 (8)	−0.0086 (6)	−0.0053 (6)	−0.0072 (6)
C24	0.0253 (7)	0.0329 (8)	0.0392 (8)	−0.0097 (6)	−0.0108 (6)	0.0093 (6)
C25	0.0283 (7)	0.0475 (10)	0.0373 (9)	−0.0137 (7)	−0.0104 (6)	0.0057 (7)
C26	0.0224 (7)	0.0436 (9)	0.0349 (8)	−0.0123 (6)	−0.0091 (6)	0.0047 (6)
C27	0.0264 (7)	0.0459 (9)	0.0331 (8)	−0.0107 (7)	−0.0079 (6)	−0.0004 (7)
C28	0.0272 (7)	0.0412 (9)	0.0389 (9)	−0.0113 (7)	−0.0093 (6)	0.0020 (7)
N1	0.0415 (7)	0.0314 (7)	0.0225 (6)	−0.0164 (5)	−0.0090 (5)	0.0023 (5)
N2	0.0295 (7)	0.0435 (8)	0.0228 (6)	−0.0085 (6)	−0.0070 (5)	−0.0016 (5)
N3	0.0306 (6)	0.0311 (7)	0.0263 (6)	−0.0045 (5)	−0.0089 (5)	0.0046 (5)
O1	0.0497 (7)	0.0647 (8)	0.0289 (6)	−0.0152 (6)	−0.0086 (5)	−0.0086 (6)
O2	0.0622 (8)	0.0773 (9)	0.0300 (6)	−0.0324 (7)	−0.0226 (6)	0.0168 (6)
O3	0.0424 (6)	0.0386 (6)	0.0321 (6)	−0.0085 (5)	−0.0082 (5)	−0.0078 (5)
O4	0.0288 (6)	0.0954 (11)	0.0531 (8)	−0.0150 (6)	−0.0005 (6)	−0.0288 (7)
O5	0.0263 (6)	0.0604 (8)	0.0531 (7)	−0.0146 (5)	−0.0095 (5)	−0.0155 (6)
O6	0.0265 (6)	0.0708 (9)	0.0519 (8)	−0.0039 (6)	−0.0068 (5)	−0.0122 (6)
O7	0.0282 (5)	0.0403 (6)	0.0514 (7)	−0.0136 (5)	−0.0183 (5)	0.0077 (5)
O8	0.0606 (8)	0.0560 (8)	0.0367 (7)	−0.0143 (6)	−0.0146 (6)	0.0056 (6)
O9	0.0783 (10)	0.0429 (8)	0.0424 (8)	−0.0081 (7)	−0.0208 (7)	0.0090 (6)

### Geometric parameters (Å, °)

**Table:**

F1—C6	1.3576 (16)	C15—H15A	0.9700
C1—O2	1.217 (2)	C15—H15B	0.9700
C1—O1	1.321 (2)	C16—N1	1.4604 (18)
C1—C2	1.479 (2)	C16—H16A	0.9700
C2—C10	1.370 (2)	C16—H16B	0.9700
C2—C3	1.426 (2)	C17—O4	1.2012 (18)
C3—O3	1.2607 (18)	C17—O5	1.3225 (18)
C3—C4	1.4490 (19)	C17—C18	1.5025 (19)
C4—C9	1.402 (2)	C18—C19	1.387 (2)
C4—C5	1.404 (2)	C18—C23	1.387 (2)
C5—C6	1.349 (2)	C19—C20	1.388 (2)
C5—H5A	0.9300	C19—H19A	0.9300
C6—C7	1.413 (2)	C20—C21	1.389 (2)
C7—C8	1.391 (2)	C20—H20A	0.9300
C7—N1	1.3936 (17)	C21—C22	1.383 (2)
C8—C9	1.4023 (19)	C21—C24	1.5162 (19)
C8—H8A	0.9300	C22—C23	1.389 (2)
C9—N3	1.4020 (18)	C22—H22A	0.9300
C10—N3	1.3397 (18)	C23—H23A	0.9300
C10—H10A	0.9300	C24—O6	1.2388 (18)
C11—N3	1.4865 (19)	C24—O7	1.2766 (18)
C11—C12	1.513 (3)	C25—O8	1.2048 (19)
C11—H11A	0.9700	C25—O9	1.324 (2)
C11—H11B	0.9700	C25—C26	1.493 (2)
C12—H12A	0.9600	C26—C27	1.390 (2)
C12—H12B	0.9600	C26—C28^i^	1.396 (2)
C12—H12C	0.9600	C27—C28	1.383 (2)
C13—N1	1.4663 (18)	C27—H27A	0.9300
C13—C14	1.511 (2)	C28—C26^i^	1.396 (2)
C13—H13A	0.9700	C28—H28	1.00 (2)
C13—H13B	0.9700	N2—H2A	0.95 (2)
C14—N2	1.486 (2)	N2—H2B	0.90 (2)
C14—H14A	0.9700	O1—H1A	1.01 (3)
C14—H14B	0.9700	O5—H5B	0.97 (3)
C15—N2	1.4854 (19)	O9—H9A	0.87 (2)
C15—C16	1.511 (2)		
			
O2—C1—O1	121.77 (15)	H15A—C15—H15B	108.0
O2—C1—C2	122.66 (16)	N1—C16—C15	110.42 (12)
O1—C1—C2	115.55 (15)	N1—C16—H16A	109.6
C10—C2—C3	120.06 (13)	C15—C16—H16A	109.6
C10—C2—C1	118.91 (14)	N1—C16—H16B	109.6
C3—C2—C1	120.95 (14)	C15—C16—H16B	109.6
O3—C3—C2	122.78 (13)	H16A—C16—H16B	108.1
O3—C3—C4	121.79 (13)	O4—C17—O5	122.82 (14)
C2—C3—C4	115.42 (13)	O4—C17—C18	123.26 (14)
C9—C4—C5	118.25 (12)	O5—C17—C18	113.87 (13)
C9—C4—C3	121.91 (13)	C19—C18—C23	119.20 (13)
C5—C4—C3	119.84 (13)	C19—C18—C17	121.79 (13)
C6—C5—C4	120.33 (13)	C23—C18—C17	118.96 (13)
C6—C5—H5A	119.8	C18—C19—C20	120.29 (13)
C4—C5—H5A	119.8	C18—C19—H19A	119.9
C5—C6—F1	117.76 (12)	C20—C19—H19A	119.9
C5—C6—C7	123.34 (13)	C19—C20—C21	120.57 (14)
F1—C6—C7	118.88 (12)	C19—C20—H20A	119.7
C8—C7—N1	122.74 (13)	C21—C20—H20A	119.7
C8—C7—C6	116.28 (12)	C22—C21—C20	119.00 (12)
N1—C7—C6	120.84 (12)	C22—C21—C24	121.62 (13)
C7—C8—C9	121.46 (13)	C20—C21—C24	119.35 (13)
C7—C8—H8A	119.3	C21—C22—C23	120.57 (13)
C9—C8—H8A	119.3	C21—C22—H22A	119.7
N3—C9—C4	118.79 (12)	C23—C22—H22A	119.7
N3—C9—C8	120.97 (12)	C18—C23—C22	120.33 (14)
C4—C9—C8	120.22 (13)	C18—C23—H23A	119.8
N3—C10—C2	124.37 (14)	C22—C23—H23A	119.8
N3—C10—H10A	117.8	O6—C24—O7	125.16 (13)
C2—C10—H10A	117.8	O6—C24—C21	117.30 (13)
N3—C11—C12	112.40 (13)	O7—C24—C21	117.54 (13)
N3—C11—H11A	109.1	O8—C25—O9	123.17 (15)
C12—C11—H11A	109.1	O8—C25—C26	123.95 (15)
N3—C11—H11B	109.1	O9—C25—C26	112.88 (14)
C12—C11—H11B	109.1	C27—C26—C28^i^	119.59 (15)
H11A—C11—H11B	107.9	C27—C26—C25	118.64 (14)
C11—C12—H12A	109.5	C28^i^—C26—C25	121.76 (15)
C11—C12—H12B	109.5	C28—C27—C26	120.27 (15)
H12A—C12—H12B	109.5	C28—C27—H27A	119.9
C11—C12—H12C	109.5	C26—C27—H27A	119.9
H12A—C12—H12C	109.5	C27—C28—C26^i^	120.14 (15)
H12B—C12—H12C	109.5	C27—C28—H28	120.3 (11)
N1—C13—C14	109.50 (12)	C26^i^—C28—H28	119.5 (11)
N1—C13—H13A	109.8	C7—N1—C16	117.61 (12)
C14—C13—H13A	109.8	C7—N1—C13	119.55 (11)
N1—C13—H13B	109.8	C16—N1—C13	110.22 (11)
C14—C13—H13B	109.8	C15—N2—C14	111.24 (11)
H13A—C13—H13B	108.2	C15—N2—H2A	108.2 (11)
N2—C14—C13	109.94 (13)	C14—N2—H2A	114.5 (11)
N2—C14—H14A	109.7	C15—N2—H2B	109.8 (13)
C13—C14—H14A	109.7	C14—N2—H2B	106.8 (12)
N2—C14—H14B	109.7	H2A—N2—H2B	106.1 (15)
C13—C14—H14B	109.7	C10—N3—C9	119.45 (12)
H14A—C14—H14B	108.2	C10—N3—C11	118.90 (12)
N2—C15—C16	111.36 (12)	C9—N3—C11	121.65 (11)
N2—C15—H15A	109.4	C1—O1—H1A	105.0 (15)
C16—C15—H15A	109.4	C17—O5—H5B	111.4 (13)
N2—C15—H15B	109.4	C25—O9—H9A	112.3 (15)
C16—C15—H15B	109.4		
			
O2—C1—C2—C10	−7.3 (2)	C18—C19—C20—C21	0.4 (2)
O1—C1—C2—C10	171.42 (13)	C19—C20—C21—C22	−2.0 (2)
O2—C1—C2—C3	176.10 (13)	C19—C20—C21—C24	176.19 (13)
O1—C1—C2—C3	−5.2 (2)	C20—C21—C22—C23	1.6 (2)
C10—C2—C3—O3	−178.58 (13)	C24—C21—C22—C23	−176.53 (14)
C1—C2—C3—O3	−2.0 (2)	C19—C18—C23—C22	−1.9 (2)
C10—C2—C3—C4	0.41 (19)	C17—C18—C23—C22	175.66 (14)
C1—C2—C3—C4	176.99 (12)	C21—C22—C23—C18	0.3 (2)
O3—C3—C4—C9	177.89 (12)	C22—C21—C24—O6	−178.99 (14)
C2—C3—C4—C9	−1.10 (19)	C20—C21—C24—O6	2.9 (2)
O3—C3—C4—C5	−1.3 (2)	C22—C21—C24—O7	1.6 (2)
C2—C3—C4—C5	179.68 (12)	C20—C21—C24—O7	−176.52 (13)
C9—C4—C5—C6	1.4 (2)	O8—C25—C26—C27	−3.9 (2)
C3—C4—C5—C6	−179.33 (12)	O9—C25—C26—C27	175.87 (13)
C4—C5—C6—F1	175.05 (11)	O8—C25—C26—C28^i^	177.53 (15)
C4—C5—C6—C7	−3.0 (2)	O9—C25—C26—C28^i^	−2.7 (2)
C5—C6—C7—C8	1.4 (2)	C28^i^—C26—C27—C28	−0.5 (2)
F1—C6—C7—C8	−176.64 (12)	C25—C26—C27—C28	−179.12 (13)
C5—C6—C7—N1	177.13 (13)	C26—C27—C28—C26^i^	0.5 (2)
F1—C6—C7—N1	−0.92 (19)	C8—C7—N1—C16	4.3 (2)
N1—C7—C8—C9	−173.88 (12)	C6—C7—N1—C16	−171.11 (13)
C6—C7—C8—C9	1.8 (2)	C8—C7—N1—C13	−133.83 (14)
C5—C4—C9—N3	179.84 (12)	C6—C7—N1—C13	50.73 (19)
C3—C4—C9—N3	0.61 (19)	C15—C16—N1—C7	158.69 (12)
C5—C4—C9—C8	1.64 (19)	C15—C16—N1—C13	−59.52 (16)
C3—C4—C9—C8	−177.59 (12)	C14—C13—N1—C7	−157.19 (13)
C7—C8—C9—N3	178.57 (12)	C14—C13—N1—C16	61.86 (16)
C7—C8—C9—C4	−3.3 (2)	C16—C15—N2—C14	−53.27 (17)
C3—C2—C10—N3	0.8 (2)	C13—C14—N2—C15	55.27 (16)
C1—C2—C10—N3	−175.81 (13)	C2—C10—N3—C9	−1.4 (2)
N1—C13—C14—N2	−59.34 (17)	C2—C10—N3—C11	179.53 (13)
N2—C15—C16—N1	55.06 (16)	C4—C9—N3—C10	0.64 (19)
O4—C17—C18—C19	174.08 (16)	C8—C9—N3—C10	178.82 (12)
O5—C17—C18—C19	−3.4 (2)	C4—C9—N3—C11	179.68 (12)
O4—C17—C18—C23	−3.4 (2)	C8—C9—N3—C11	−2.1 (2)
O5—C17—C18—C23	179.09 (14)	C12—C11—N3—C10	98.46 (16)
C23—C18—C19—C20	1.5 (2)	C12—C11—N3—C9	−80.58 (17)
C17—C18—C19—C20	−175.97 (14)		

Symmetry codes: (i) −*x*+2, −*y*, −*z*+1.

### Hydrogen-bond geometry (Å, °)

**Table:**

*D*—H···*A*	*D*—H	H···*A*	*D*···*A*	*D*—H···*A*
C15—H15B···O1^ii^	0.97	2.38	3.274 (2)	153
O5—H5B···O7^iii^	0.97 (3)	1.70 (3)	2.6648 (15)	169 (2)

Symmetry codes: (ii) −*x*+1, −*y*, −*z*+1; (iii) *x*−1, *y*, *z*.
